# Insult of Ultraendurance Events on Blood Pressure: A Systematic Review and Meta-Analysis

**DOI:** 10.7759/cureus.46801

**Published:** 2023-10-10

**Authors:** Steven B Hammer, Fred Strale Jr., Timothy B Williams, Shantele L Kemp Van Ee, James W Agnew

**Affiliations:** 1 Anatomy and Physiology, Indian River State College, Fort Pierce, USA; 2 Statistics, Wayne State University, Detroit, USA; 3 Medicine, Dr. Kiran C. Patel College of Osteopathic Medicine, Nova Southeastern University, Fort Lauderdale, USA

**Keywords:** orthostasis, blood pressure, ultrarunning, ultramarathon, ultraendurance

## Abstract

The rise of ultraendurance sports in the past two decades warrants evaluation of the impact on the heart and vessels of a growing number of athletes participating. Blood pressure is a simple, inexpensive method to evaluate one dimension of an athlete’s cardiovascular health. No systematic review or meta-analysis to date has chronicled and delineated the effects of ultraendurance races, such as ultramarathons, marathons, half-marathons, and Ironman triathlon events, specifically on heart rate (HR), systolic blood pressure (SBP), diastolic blood pressure (DBP), pulse pressure (PP), and mean arterial pressure (MAP) measurements in supine and standing positions before and after the event. This meta-analysis reviews the effects of ultraendurance events on positional and calculated hemodynamic values. Data were extracted from 38 studies and analyzed using a random effects model with a total of 1,645 total blood pressure measurements. Of these, 326 values were obtained from a standing position, and 1,319 blood pressures were taken supine. Pre-race and post-race measurements were evaluated for clinical significance using established standards of hypotension and orthostasis. HR and calculated BP features, such as PP and MAP, were evaluated. Across all included studies, the mean supine post-race HR increased by 21±8 beats per minute (bpm) compared to pre-race values. The mean standing post-race HR increased by 23±14 bpm when compared with pre-race HR. Overall, there was a mean SBP decrease of 19±9 mmHg and a DBP decrease of 9±5 mmHg post-race versus pre-race values. MAP variations reflected SBP and DBP changes. The mean supine and standing pre-race blood pressures across studies were systolic (126±7; 124±14) and diastolic (76±6; 75±12), suggesting that some athletes may enter races with existing hypertension. The post-race increase in the mean HR and decline in mean blood pressure across examined studies suggest that during long-term events, ultramarathon athletes perform with relatively asymptomatic hypotension.

## Introduction and background

The effects of exercise on cardiovascular function are well known, with years of observable study focused on various parameters of cardiovascular health [1]. Recent years have seen an increase in the popularity of marathon events exceeding six hours, requiring advanced training and strength, dubbed "ultraendurance" [2]. It is reasonable to postulate that ultraendurance athletes are typically healthier and possess a lower risk for hypertension and heart disease due to consistent training between events. However, as the population of ultraendurance athletes increases in size and variety, this paradigm is changing. According to Ronto’s 2020 study of long-distance running, participation in ultramarathons alone has increased by 1676% since 1996, up to 611,098 runners with an average age of 42.3 years [3]. 

The stress on the vasculature to maintain afterload against loss of volume during lengthy and strenuous exercise has been noted to cause hypotension during and after ultraendurance events [4]. Extreme long-distance events and prolonged cardiac exertion are associated with ventricular dysfunction in athletes over time [5,6]. Ultraendurance sports may offer limited cardiovascular health benefits due to exhaustion of cardiac and vascular endothelial compensatory mechanisms [7].

Most published studies on cardiovascular health in ultraendurance athletes utilize modalities such as electrocardiogram or echocardiogram to collect data on physiological response, with blood pressure (BP) data captured as a secondary or tertiary metric. Therefore, a lack of specific results exists on how the BP of endurance athletes changes after the event, particularly considering challenges to orthostasis. 

A fundamental discussion on ultraendurance athletes’ pre-race BP has not been addressed, including the incidence of cardiovascular disease in this increasingly heterogeneous population. Additionally, many studies only record the BP in a supine position. This standard method may create inaccuracies in BP measurement in athletes who spend 20 or 30 hours standing, potentially obscuring hypotensive results. This study aims to provide comprehensive data analysis on the BP measurements in ultraendurance athletes before and after sporting events to outline the risk for hypotension-related conditions such as brain ischemia, myocardial infarction, or acute kidney injury.

## Review

Materials and methods

Search Strategy

We searched ScienceDirect, PubMed (Medline), and CINAHL beginning with inclusion and exclusion criteria. Articles in English containing mean BP values taken in a standing and/or supine position, with standard deviations, taken from participants that completed ultraendurance races. Sports not involved with running, swimming, or cycling and did not include BP data were excluded. Races were 21 kilometers or more, with a six-hour or greater duration. No other systematic reviews, review papers, case reports, or book chapters were included in this analysis. 

Thirty-eight studies were analyzed using a random effects model with 1,645 measurements from research subjects. There were 326 measurements in a standing position and 1,319 in a supine position. These studies mainly focused on BP values and the effects ultraendurance events had on them. For each of the 38 included studies, sample size (n), mean, standard deviation, standard error, 95% confidence interval, z-value, p-value, effect size (unstandardized mean differences), standardized measure of effect size (Hedges’ g), and relative weights for pre-race to post-race heart rate (HR), systolic blood pressure (SBP), diastolic blood pressure (DBP), pulse pressure (PP) and mean arterial pressure (MAP) were computed using their respective formulas.

**Figure 1:**
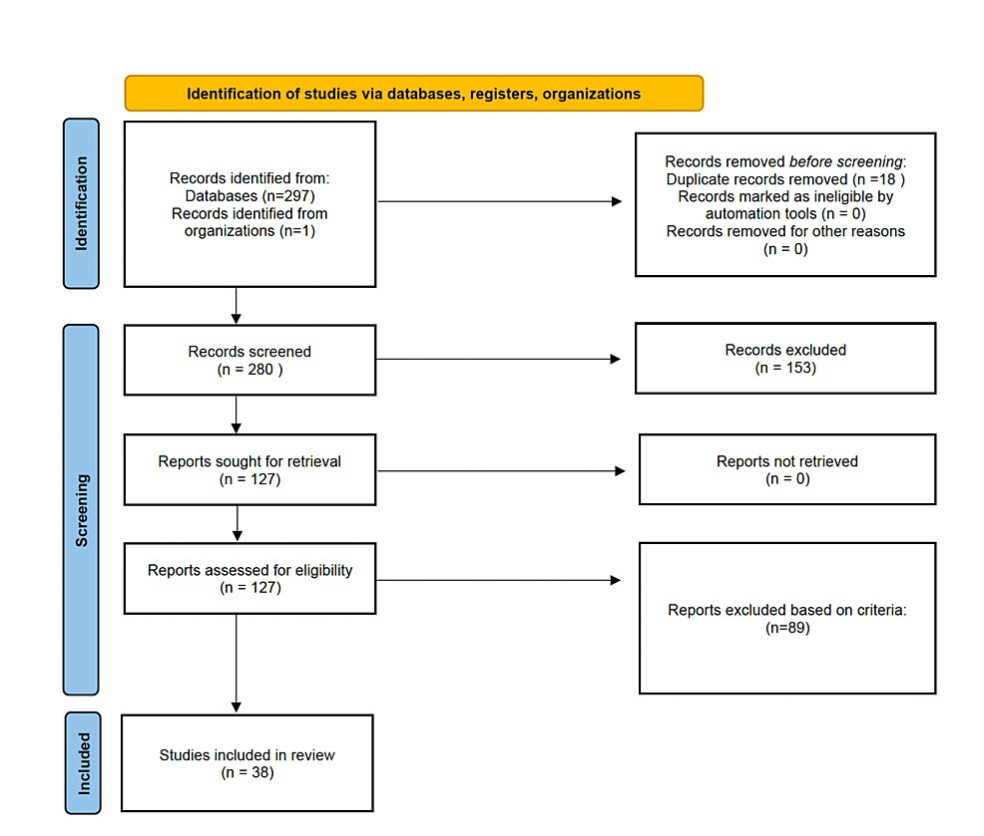
PRISMA Outline of the Selection Process. PRISMA: Preferred Reporting Items for Systematic Reviews and Meta-Analyses

Using the “Advanced Search” feature in each database, the following Boolean string was entered: ultramarathon OR ultraendurance OR ultrarunning OR mountain endurance AND blood pressure. See the Preferred Reporting Items for Systematic Reviews and Meta-Analyses (PRISMA) chart in Figure 1. The search results were then imported into Rayyan for screening.

Selection Process

After completing the searches, 297 results were selected. After removing duplicates using automated processes, 279 full-text articles were accessed through their respective journals. Each article was screened independently by three reviewers to determine eligibility according to the preset inclusion criteria. Only records that met all of the inclusion criteria and none of the exclusion criteria were selected. Of the 279 articles in the results, 38 studies were selected.

Data Items

Reported mean pre-race and post-race HR, SBP, DBP, PP, and MAP were documented in all included studies. In addition, variables such as location, setting, gender, temperature, humidity, altitude, distance, and time were documented and are detailed in Table 1. For studies that did not record PP or MAP, they were calculated using the following formulas: MAP = DBP + 1/3(SBP - DBP); PP = SBP - DBP. Standard deviations for these unlisted data were calculated from the mean standard deviation of the systolic and/or diastolic blood pressures.

**Table 1:** Study Characteristics, Demographics, and Conditions. *Hammer S, Strale F, Williams T, Kemp Van Ee SL, Agnew J. Poster Presentation: Effects of Ultraendurance Running on Athlete Heart Rate and Blood Pressure, presented at International Society of Sports Nutrition 20th Annual National Conference on June 15, 2023 in Fort Lauderdale, Florida.

Study	n=	Location	Gender	Conditions	Altitude	Distance/Time
Belinchon-deMiguel et al., 2019 [8]	46	Mountains, Spain	Not stated	Not stated	740-1200 m	100,112 km
Belinchon-deMiguel et al., 2018 [9]	11	Mountains, Spain	Not stated	Not stated	1020-1920 m	51.2 km
Benedetti et al., 2021 [10]	22	Flat trail, Italy	12 male 10 female	5-12° C, 76-100% humidity	57.9 km	24-hour ultra
Bonsignore et al., 2017 a [11]	21	Mountains, Canada	15 male 6 female	8.7-26.3° C	500-2200 m	80 km
Bonsignore et al., 2017 b [11]	25	Mountains, Canada	22 male 3 female	8.7-26.3° C	600-2300 m	195 km
Burr et al., 2012 a [12]	15	Mountains, British Columbia	8 male 7 female	6-30° C	600-2300 m	120 km
Burr et al., 2012 b [12]	11	Mountains, British Columbia	9 male 2 female	6-30° C	600-2300 m	195 km
Christou et al., 2020 [13]	27	Mountains, Greece	19 male 8 female	13-22° C	0-1200 m	246 km
D’Silva et al., 2020 [14]	68	England	36 male 32 female	Not stated	11 m	42.2 km London Marathon
Deiseroth et al., 2018 [15]	47	Mountains, Germany	All male	Not stated	520 m	42.2 km
Douglas et al., 1987 [16]	21	Triathlon, Hawaii	13 male 8 female	24-42° C, 40-80% humidity	0-2079 m	2.4 mile swim 112 mile bike 42.2 km run
Dávila-Románet al., 1997 [17]	14	Mountains, Colorado	Not stated	2-24° C, 10-60% humidity	2350-4300 m	163 km
Faconti et al., 2020 [18]	25	England	17 male 8 female	8-12° C	11 m	42.2 km
Gratze et al., 2005 [19]	25	Triathlon, Austria	All male	Not stated	446 m	3.9 m swim 180 km bike 42.2 km run
Hammer et al., 2023*	217	Trail, Florida	136 male 81 female	26.5-30° C, 65% humidity	0-10 m	50, 80, 100, 120, 160, 240 km
Holtzhausen et al., 1995 [20]	31	Trail, South Africa	Not stated	14-25° C, 24-44% humidity	655 m	80 km
Kalliokoski et al., 2004 [21]	7	Marathon, Finland	All male	Not stated	59 m	42.2 km
Koutlianos et al., 2021 [22]	20	Mountains, Greece	Not stated	13-22° C	0-1200 m	246 km
Krzeminski et al., 2016 [23]	9	Poland	All male	12-24° C, 55-60% humidity	590 m	100 km
Lucas et al., 2008 [24]	9	New Zealand	All male	Not stated	16 m	24 hour ~ 200 km
Malek et al., 2020 [25]	18	Outdoor Road, Poland	15 male 3 female	Not stated	100 m	100 km
Martinez-Navarro et al., 2018 [26]	50	Mountain Trail, Spain	44 male 6 female	Not stated	40-1280 m	118 km
Mauget-Faysse et al., 2021 [27]	29	Road Marathon, France	Not stated	Not stated	35 m	42.2 km
Menicucci et al., 2013 [28]	20	Triathlon, Italy	Not stated	Not stated	14 m	3.8 km, swim 180 km cycle, 42.2 km run
Mourot et al., 2020 [29]	25	Outdoor road, Italy	12 male 13 female	19° C 71%	59 m	21 km, half marathon
Murrell et al., 2007 [30]	9	New Zealand	5 male 4 female	Not stated	525-837 m	42 km
Neilan et al., 2006 [31]	60	Massachusetts	41 male 19 female	Not stated	43 m	42.2 km, Boston Marathon
Neilan et al., 2006 [32]	20	Massachusetts	Not stated	Not stated	43 m	42.2 km
Niemela et al., 1984 [33]	13	Outdoor track, Finland	All male	7-15° C 50-60%	15 m	114-214 km, 24 hours
Oxborough et al., 2006 [5]	35	Road, England	29 male 6 female	Not stated	11 m	42.2 km, London Marathon
Paleczny et al., 2021 [34]	25	Road Marathon, Poland	All male	Not stated	130 m	42.2 km
Parsons et al., 2021 [35]	24	Road Marathon, England	Not stated	8-12° C 49-78%	11 m	42.2 km
Passaglia et al., 2013 [36]	12	Outdoor track, Brazil	All male	9-21° C	935 m	111-169 km, 24 hours
Privett et al., 2010 [37]	10	Outdoor road, England	All male	20-24° C	11 m	42 km
Roeh et al., 2019 [38]	212	Road Marathon, Germany	All male	Not stated	520 m	42.2 km
Scott et al., 2009 [39]	35	Mountain, California	25 male 10 female	Not stated	2066 m	160 km
Taksaudom et al., 2017 [40]	33	Mountain trail, Thailand	Not stated	Not stated	3400 m	66 km
Trullàs et al., 2018 [41]	21	Mountains, Spain	All male	Not stated	3000 m	45 km

Effect Measures

Effect sizes relative to the difference between pre-race and post-race measurements were reported as the unstandardized mean difference (UMD) and the Hedges’ g (a measure of effect size) in the synthesis and presentation of results to better appreciate the practical significance of the magnitude of difference between pre-race and post-race measurements.

Data Collection and Synthesis Methods

Study intervention characteristics included ultraendurance events such as ultramarathons, marathons, half-marathons, and Ironman triathlons. The relevant data was collected into a shared Microsoft Excel spreadsheet and imported into IBM SPSS Statistics for Windows, Version 28 (Released 2021; IBM Corp., Armonk, New York, United States). Records were later checked for accuracy by two additional independent reviewers.

The participants’ position during BP measurement was also recorded as standing or supine. When not noted in the study, the reviewers assumed the measurements were taken with the participant in a supine position, as recorded during echocardiograms. HR, SBP, and DBP were collected as mean +/- standard deviation. MAP was recorded as mean MAP +/- standard deviation or calculated from the mean SBP and DBP, using the standard formula previously detailed. Mean PP was also recorded directly or calculated from the mean SBP and DBP, using the formula listed above. Standard deviations for calculated data were computed from the mean of the systolic and diastolic pressure standard deviation. No automation tools were used in the data collection process. 

Data were further divided into supine or standing values for proper comparisons. Pre-race and post-race supine and standing data were extracted from the 38 relevant studies and placed into an Excel spreadsheet, which was then pasted into IBM SPSS Statistics for Windows, Version 28. The 38 selected studies were analyzed on HR, SBP, DBP, PP, and MAP using a random effects model. The following values were then reported in a table format: study name, study year, effect sizes, standard errors, z-scores, p-values, 95% confidence intervals, random effects weights, and random effects weight percentage. SBP data were reported in Tables 2, 3.

**Table 2:** Standing Systolic Blood Pressure Statistics Across Studies Before and After the Race. *Hammer S, Strale F, Williams T, Kemp Van Ee, SK, Agnew J. Poster Presentation: Effects of Ultraendurance Running on Athlete Heart Rate and Blood Pressure. Presented at the International Society of Sports Nutrition 20th Annual National Conference on June 15, 2023 in Fort Lauderdale, Florida.

Study Name	n=	Lower Limit (mmHg)	Upper Limit (mmHg)	z-Value	p-Value	SE	Difference in Means (mmHg)	Hedges’ g	Relative Weight
Hammer et al., 2023*	217	18.60	23.40	17.16	0.0000	1.22	21	1.64	29.47
Holtzhausen et al., 1995 [20]	31	26.03	37.97	10.50	0.0000	3.05	32	2.63	15.71
Lucas et al., 2008 [24]	9	2.05	33.95	2.21	0.0270	8.14	18	0.99	10.09
Martinez-Navarro et al., 2018 [26]	50	12.70	23.30	6.66	0.0000	2.70	18	1.32	22.80
Murrell et al., 2007 [30]	9	0.90	35.10	2.06	0.0391	8.72	18	0.93	10.20
Privett et al., 2010 [37]	10	-13.49	23.49	0.53	0.5961	9.43	5	0.23	11.73
Group Statistics Standing	326	16.52	26.33	8.56	0.0000	2.50	21	1.41	
Overall Statistics Standing and Supine	1753	5.89	26.11	3.10	0.0019	5.16	16	1.11	

**Table 3:** Supine Systolic Blood Pressure Statistics Across Studies Before and After the Race. *Hammer S, Strale F, Williams T, Kemp Van Ee, SK, Agnew J. Poster Presentation: Effects of Ultraendurance Running on Athlete Heart Rate and Blood Pressure. Presented at the International Society of Sports Nutrition 20th Annual National Conference on June 15, 2023 in Fort Lauderdale, Florida.

Study Name	n=	Lower Limit (mmHg)	Upper Limit (mmHg)	z-Value	p-Value	SE	Difference in Means (mmHg)	Hedges’ g	Relative Weight
Belinchón-deMiguel et al., 2019 [8]	46	11.89	22.11	6.52	0.0000	2.61	17	1.35	3.42
Belinchón-deMiguel et al., 2018 [9]	11	8.30	31.70	3.35	0.0008	5.97	20	1.37	1.67
Benedetti et al., 2021 [10]	22	4.73	21.27	3.08	0.0021	4.22	13	0.91	2.67
Bonsignore et al., 2017 a [11]	25	1.80	14.20	2.53	0.0114	3.16	8	0.70	2.88
Bonsignore et al., 2017 b [11]	21	-3.22	13.22	1.19	0.2329	4.19	5	0.36	2.72
Burr et al., 2012 a & b [12]	26	2.83	13.17	3.03	0.0024	2.64	8	0.83	2.90
Christou et al., 2020 [13]	27	-0.34	18.34	1.89	0.0589	4.76	9	0.51	3.01
D’Silva et al., 2020 [14]	68	-0.03	8.03	1.94	0.0519	2.06	4	0.33	3.99
Deiseroth et al., 2018 [15]	47	6.94	17.06	4.65	0.0000	2.58	12	0.95	3.55
Douglas et al., 1987 [16]	21	-3.22	13.22	1.19	0.2329	4.19	5	0.36	2.72
Faconti et al., 2020 [18]	25	4.46	15.54	3.54	0.0004	2.83	10	0.98	2.81
Gratze et al., 2005 [19]	25	3.12	10.88	3.54	0.0004	1.98	7	0.98	2.81
Hammer et al., 2023*	217	12.53	17.47	11.88	0.0000	1.26	15	1.14	4.61
Holtzhausen et al., 1995 [20]	31	1.78	16.22	2.44	0.0146	3.69	9	0.61	3.16
Kalliokoski et al., 2004 [21]	7	-5.23	23.23	1.24	0.2151	7.26	9	0.62	1.37
Koutlianos et al., 2021 [22]	20	-1.65	17.65	1.62	0.1043	4.92	8	0.50	2.64
Krzemiński et al., 2016 [23]	9	6.91	29.09	3.18	0.0015	5.66	18	1.43	1.43
Lucas et al., 2008 [24]	9	-2.33	18.33	1.52	0.1290	5.27	8	0.68	1.63
Martinez-Navarro et al., 2018 [26]	50	1.06	10.94	2.38	0.0172	2.52	6	0.47	3.70
Mauget-Faysse et al., 2021 [27]	29	2.01	15.99	2.52	0.0116	3.57	9	0.65	3.07
Menicucci et al., 2013 [28]	20	11.87	26.13	5.22	0.0000	3.64	19	1.62	2.31
Mourot et al., Female, 2020 [29]	13	0.71	15.29	2.15	0.0316	3.72	8	0.82	2.03
Mourot et al., Male, 2020 [29]	12	2.05	19.95	2.41	0.0160	4.56	11	0.95	1.90
Murrell et al., 2007 [30]	9	2.03	21.97	2.36	0.0184	5.09	12	1.06	1.54
Neilan et al., 2006 [31]	60	5.14	14.86	4.03	0.0001	2.48	10	0.73	3.84
Neilan et al., 2006 [32]	20	5.48	18.52	3.61	0.0003	3.32	12	1.12	2.49
Niemela et al., 1984 [33]	13	18.70	35.30	6.38	0.0000	4.23	27	2.42	1.47
Oxborough et al., 2006 [5]	35	4.07	13.93	3.58	0.0003	2.51	9	0.85	3.25
Paleczny et al., 2021 [34]	25	4.96	25.04	2.93	0.0034	5.12	15	0.82	2.85
Parsons et al., 2021 [35]	24	3.38	22.62	2.65	0.0081	4.91	13	0.75	2.82
Passaglia et al., 2013 [36]	20	13.00	23.00	7.06	0.0000	2.55	18	2.19	2.07
Privett et al., 2010 [37]	10	-2.08	40.08	1.77	0.0773	10.76	19	0.76	1.73
Roeh et al., 2019 [38]	212	4.41	9.59	5.31	0.0000	1.32	7	0.51	4.65
Scott et al., 2009 [39]	160	2.58	7.42	4.05	0.0001	1.23	5	0.45	4.54
Taksaudom et al., 2017 [40]	33	9.65	22.35	4.94	0.0000	3.24	16	1.20	3.08
Trullàs et al., 2018 [41]	25	10.93	25.07	4.99	0.0000	3.61	18	1.39	2.68
Group Statistics Supine	1427	9.29	12.91	12.02	0.0000	0.92	11	0.87	
Overall Statistics Standing and Supine	1753	5.89	26.11	3.10	0.0019	5.16	16	1.11	

Results

Overall changes were reported as mean ± standard deviation (Tables 4, 5), which were calculated from all included studies. In a supine position, the mean HR across studies increased in the post-race measure by 21±8 bpm, consistent with catecholamine release during sustained exercise. Though mean HRs while standing were on average higher pre-race than those recorded supine during the same time interval, the quantity of increase was similar after the race between supine and standing positions. Across all studies, there were small, but notable decreases in mean standing blood pressure post-race compared to values recorded before the race while standing. These changes are similar to those observed in the supine position before and after the races. These values may reflect the compensatory sympathetic response necessary to account for any hypovolemia via insensible losses intra-race. These numbers demonstrate the degree to which the body required compensation to maintain adequate perfusion with fluid loss.

**Table 4:** Blood Pressure Changes Across Included Studies in the Standing Position. Reported as mean and standard deviation calculated from all studies referenced in Table 2. Note that negative pre-race to post-race change values represent an overall increase and positive values represent an overall decrease. The p-value set at p<0.05, comparisons are for pre-race to post-race changes. HR: Heart rate; SBP: systolic blood pressure; DBP: diastolic blood pressure; PP: pulse pressure; MAP: mean arterial pressure

Standing	HR	SBP	DBP	PP	MAP
Pre-race mean	75±8	124±14	75±12	50±6	90±12
Post-race mean	98±11	106±17	65±12	40±10	76±10
Pre-race to post-race change	-23±14	19±9	9±5	9±5	14±4
p-value	p<0.001	p<0.001	p<0.001	p<0.001	p<0.001
n= (sample size)	6	6	6	6	6

**Table 5:** Blood Pressure Changes Across Included Studies in the Supine Position. Reported as mean and standard deviation calculated from studies referenced in Table 3. Note that negative pre-race to post-race change values represent an overall increase and positive values represent an overall decrease. The p-value set at p<0.05, comparisons are for pre-race to post-race changes.

Supine	HR	SBP	DBP	PP	MAP
Pre-race mean	61±4	126±7	76±6	51±6	91±6
Post-race mean	81±9	115±7	71±5	44±6	85±6
Pre-race to post-race change	-21±8	12±5	5±4	7±5	7±4
p-value	p<0.001	p<0.001	p<0.001	p<0.001	p<0.001
n= (sample size)	34	36	34	33	33

Discussion

Heart Rate

Overall, the results demonstrated a mean increase in the pre-race to post-race supine HR of 21±8 bpm and a mean increase in pre-race to post-race standing HR of 23±14 bpm. This is consistent with the effects of physical exertion and is commonly observed in athletes within one hour of completing a race [8,42]. Six studies that measured supine and standing BP in an orthostatic BP test were also compared. The pre-race supine-to-standing mean difference was 14 bpm or a 23% increase pre-race. The post-race supine-to-standing mean difference was 17 bpm or a 21% increase. These changes in the HR are the product of an appropriate orthostatic response, increasing by 15-20 bpm. This is a predictable rate change and does not directly reflect orthostatic hypotension [43].

Systolic and Diastolic Blood Pressure 

Overall, pre-race to post-race supine systolic blood pressure results show a mean decrease of 12±5 mmHg and a mean diastolic decline of 5±4 mmHg. However, the overall pre-race to post-race standing systolic mean decrease was 19±9 mmHg, and the diastolic mean decline was 9±5 mmHg. Orthostatic change pre-race was modest, with a systolic change of -2 mmHg or a 1.5% decline and a diastolic change of -5 mmHg, reflecting a 6.6% reduction. However, post-race changes were significant, with a mean supine systolic of 115±7 mmHg and a mean standing systolic of 106±17 mmHg. The mean diastolic supine was 71±5 mmHg, and the mean diastolic standing was 65±12 mmHg. These numbers reflect a decline of 18 mmHg, or a 14.5% drop in systolic blood pressure, and a diastolic decrease of 10 mmHg, or a 13.3% reduction. Although these changes do not meet the criteria for orthostatic hypotension, this notable drop may result from prolonged time in the standing position.

The changes in BP are consistent with post-exercise hypotension and are greater than the decreases observed in the average population [36]. Among potential mechanisms that likely drive post-exercise hypotension, volume depletion plays a significant role. All ultramarathon runners undergo body mass loss and dehydration within the first few hours of a race [44]. Although the risk of hyponatremic hypervolemia remains due to increased water intake and overhydration, several studies indicate ultraendurance athletes can maintain a euhydrated state throughout the race [45].

Pulse Pressure

Supine pre-race to post-race mean decline was 7±5 mmHg, and standing pre-race to post-race mean drop was 9±5 mmHg. The mean pre-race orthostatic pressure change (supine to standing) was a 1 mmHg decrease, or a 1.9% reduction. The mean post-race orthostatic pressure decline was 10 mmHg, representing a 20% drop. 

These changes in pulse pressure bear clinical significance. In the general population, pulse pressures of 40 mmHg are associated with a 5.6% risk of atrial fibrillation occurring in the individual’s lifetime, with a positive correlation between pulse pressure and incidence of atrial fibrillation [45]. In young endurance athletes, the occurrence is extremely low, but in veteran athletes, the risk of atrial fibrillation increases significantly with age. Additionally, the incidence of atrial fibrillation and decreased pulse pressure post-race is associated with atrial enlargement, which is well documented in ultraendurance runners [45-47]. 

*Mean Arterial Pressure* 

Average supine pre-to-post MAP decreased by 7±5 mmHg (91±6 to 85±6 mmHg). The average standing pre-to-post MAP change recorded was -14±4 (90±12 to 76±10 mmHg). These changes are expected and reflect the changes in systolic and diastolic pressures. The average pre-race supine-to-standing MAP change was -1 mmHg or a decrease of 1.1%. After the race, the average supine-to-standing MAP was -9 mmHg, for a 10.6% drop.

Due to the clinical value of MAP as a measurement of organ perfusion, particular interest was taken in analyzing this value. Average MAP changes are notable but do not reflect life-threatening pressure losses. Decreased renal perfusion in exercise is a physiological change, not necessarily an indicator of renal damage [48]. However, when considering the characteristic increase in various serum markers in this athlete population, such as the increases in creatine kinase and myoglobin, the loss of renal perfusion may contribute to renal damage and a decreased glomerular filtration rate [49,50].

Baseline Hypertension

In a meta-analysis by Pan et. al, the relative risk for sudden cardiac death in hypertensive subjects was 2.10, with a 1.28 increase per 20 mmHg rise in systolic blood pressure, and 1.09 for a 10 mmHg increase [51]. American and European guidelines for hypertension both show optimal BP as < 120/80 mmHg. Of the studies examined in this meta-analysis, mean supine and standing pressures were systolic (126±7; 124±14) and diastolic (76±6; 75±12) respectively. The upper level of the pre-race range for blood pressure indicates preexisting hypertension in this population. However, this observation is only relevant with the supporting age data from participants. Hoffman et al. reported in 2014 that the mean age of 100-mile ultramarathon runners was 44.5 years, and were predominantly male (80.2%), offering a likely explanation for the mean elevated blood pressure at baseline [52]. Kim et al. analyzed baseline blood pressure data from 571 middle-aged (the mean age was 48.8 ± 6.6), male runners and found that 241 subjects had high resting blood pressures (the mean resting SBP/DBP was 150.0 ± 10.0/93.6 ± 10.1 mmHg) [53]. These subjects were also found to have systolic blood pressures greater than 210 mmHg during exercise (the mean SBP maximum was 227.9 ± 23.6 mmHg), consistent with exercise-induced hypertension (EIH) [53]. EIH is a known risk factor for stroke, coronary artery disease, and the development of hypertension later in life [54]. In a confounding fashion, this meta-analysis found a lower mean pre-race blood pressure overall compared to Kim et. al, due to a wider range of subject ages, a variable that was not accounted for in this analysis.

Intra-race Hypotension

Thirty-two of the studies included in this analysis only reported supine blood pressures, while only six studies measured standing and supine blood pressures. Measurements obtained in both standing and supine positions provide screening for orthostasis and fluid depletion. During the post-race period, many of the runners presented with mild hypotension in the range of 90-100 mmHg systolic, carrying the risk of decreased organ perfusion and its effects, from altered mental status to acute kidney injury. Individual runners across all races chose the amount of oral intake during the event, and studies did not record the amount to better determine the adequacy of hydration.

Future Research

The growing popularity of ultraendurance events has changed the demographic of participants and understanding baseline comorbidities is paramount. Observing runners' response to extreme events in light of differing disease states may elucidate the degree of cardiovascular stress ultra long-distance running has on middle-aged athletes. Those running with baseline hypertension may be untreated or under-medicated and runner disease states and their treatment are not investigated in this study. As hypertension is common in middle-aged and older adults, the effects of an increased baseline blood pressure along with commonly prescribed antihypertensive medications on cardiovascular and neurological compensatory mechanisms interacting with the sympathetic response related to racing should be investigated.

Additional parameters for future research include the duration for which the observed changes in blood pressure last, which would require serial blood pressure measurements in ultramarathon runners. Moreover, the blood pressure reduction that occurs during ultraendurance events may be therapeutic for participants with pre-race hypertension. Quantifying sympathetic tone and activity present in ultramarathon runners pre-race may offer an explanation for the elevated blood pressures. A recent study by Vieluf et al. describes compensatory autonomic nervous system activity in a small sample (n=13) of runners during a 65-kilometer race [55]. A dedicated literature review and observational studies focusing on these topics will provide useful information for clinicians caring for ultraendurance athletes and provide education for the participants.

## Conclusions

Blood pressure is a relatively quick and inexpensive measure of cardiovascular function not requiring highly specialized skills, yet most ultraendurance events do not use this as a measure to screen athlete vascular health. These studies demonstrate many athletes experience significant fluctuations in blood pressure even while asymptomatic. With the modern increase in the average age of ultraendurance athletes, the dangers of cardiac strain and organ hypoperfusion during and after the events may be compounded by existing comorbidities affecting compensatory mechanisms. Athletes need to screen for and consider existing hypertension when selecting events. Understanding limitations will assist aging athletes in choosing safe training methods and an appropriate level of participation in ultraendurance events.
